# A commentary on ‘Clinical evaluation of balloon occlusion of the lower abdominal aorta in patients with placenta previa and previous cesarean section: a retrospective study on 43 cases’

**DOI:** 10.1097/JS9.0000000000001214

**Published:** 2024-02-21

**Authors:** Huaxiang Deng, Jianxia Zhang, Qiuyun Gao, Huiling Qiu, Guizhen Li

**Affiliations:** Department of Gynaecology, Boao Yiling Care Center, Qionghai, Hainan, People’s Republic of China

*Dear Editor*,

It was with great interest that we read the article published in the *International Journal of Surgery*. Chen *et al*.^1^ conducted a retrospective cohort analysis of patients who had placenta previa and had cesarean section (CS). The results of the trial showed that, without endangering the babies, balloon occlusion of the lower abdominal aorta appears to be an effective way to minimize postpartum hemorrhage, the need for blood transfusions, and the risk of hysterectomy.

Placenta previa is a condition in which the placenta partially or fully blocks the lower uterine cervix of the endometrial orifice, resulting in significant hemorrhage, disseminated intravascular coagulation (DIC), liver and kidney damage, and even hysterectomy. It has been noticed that the average blood loss volume in patients with placenta increta or percreta is 3000 ml; in 20% of cases, this volume surpasses 5000 ml, and in 10% of cases, it even reaches 10 000 ml. In pregnant patients with pernicious placenta previa who are undergoing CS, some intravascular interventional therapies have been used in obstetrics to reduce intraoperative blood loss, transfusion, and hysterectomy rates, including uterine artery embolization, common iliac artery balloon occlusion, prophylactic lower abdominal aorta balloon occlusion, bilateral internal iliac artery balloon occlusion, and other kinds of intervention methods^2^.

The abdominal aortic balloon occlusion (AABO) is a novel procedure used to replace hysterectomy in Pernicious placenta previa patients who want to maintain their fertility when a significant hemorrhage happens during obstetric CS^3^. Through the right femoral artery, interventional specialists place a balloon catheter into the lower part of the abdominal aorta, just beneath the renal artery entrance. After the fetus is delivered and the cord is clamped, the balloon is quickly inflated to temporarily restrict the uterine blood supply, reducing hemorrhage while the surgeon physically removes the placenta and stitches the uterine incision^4^. Several studies revealed that prophylactic temporary AABO could reduce the rate of post-operative uterine artery embolism and the need for transfusions. However, because AABO is an intrusive procedure, there is a risk of consequences such as first vessel injury, arterial thrombosis, puncture point hemorrhage, lower limb ischemia necrosis, organ and tissue reperfusion injury, acute renal failure, and infrequently, artery rupture. Yin *et al*.^3^ discovered that AABO is an effective and safe way to improve maternal outcomes for patients with placenta previa accreta without increasing problems, and it is not harmful to the newborn.

This study looked into the effectiveness of balloon occlusion of the lower abdominal aorta in CS surgery for patients with placenta previa and a previous CS. To sum up, AABO is a therapy that is increasingly being used for individuals who have placenta accrete. It has a positive impact by lowering the need for blood transfusions during or after surgery, bleeding, hysterectomy, and DIC rates. Several factors should be considered: this was a single-center and retrospective study with stringent inclusion criteria; the small sample size and lack of long-term follow-up, which may have resulted in some bias in the results; the surgery level of the surgeon, the conditions of the patients and the type of placenta previa may be the sources of bias^5^. A bigger sample size was required to undertake propensity score analysis and reduce the indication bias for the surgeon’s decision to utilize AABO. Furthermore, more complete maternal and neonatal outcomes will be evaluated.

## Ethical approval

This manuscript is a comment. Don’t need ethical approval.

## Consent

This manuscript is a comment. Don’t need patient consent.

## Author contribution

H.D. and J.Z.: study concept or design, data collection, data analysis or interpretation, and writing the paper; Q.G. and H.Q.: data analysis or interpretation; G.L.: study concept or design and writing and revising the paper.

## Conflicts of interest disclosure

This manuscript is a comment without conflicts of interest.

## Research registration unique identifying number (UIN)

This manuscript is a comment. Don’t need UIN.

## Guarantor

Guizhen Li.

## Data availability statement

This manuscript is a comment. Don’t need a Data availability statement. However, all the data from the current study are publicly available.

## Provenance and peer review

This manuscript is a comment without being invited.
